# Ankylosing spondylitis complicating Turner syndrome: Two case reports and a literature review

**DOI:** 10.1097/MD.0000000000021636

**Published:** 2020-08-14

**Authors:** Fang-Fei Chen, Xue-Han Zhang, Yang Jiao

**Affiliations:** a Department of General Internal Medicine; b Department of Health Care, Peking Union Medical College Hospital, Chinese Academy of Medical Sciences & Peking Union Medical College, Beijing, China.

**Keywords:** Turner syndrome, ankylosing spondylitis, chromosomal anomaly

## Abstract

**Rationale:**

Turner syndrome (TS) is an anomaly caused by loss of part of or all the X chromosomes. Ankylosing spondylitis (AS) is an HLA-B27-associated autoimmune disease with a male predominance. It is widely accepted that TS patients are at higher risk of autoimmune diseases, but AS in TS patients has only rarely been reported.

**Patient concerns:**

A 13-year-old TS patient presented with intermittent pain in both hip joints, and a 27-year-old TS patient presented with thoracic kyphosis and a history of AS.

**Diagnoses:**

Both patients were diagnosed with AS according to their symptoms, laboratory results, and imaging.

**Interventions:**

The first patient was treated with tocilizumab for 8 months, whereas the second patient was treated with diclofenac initially with subsequent surgery for thoracic kyphosis.

**Outcomes:**

Treatment relieved the symptoms of both patients and laboratory parameters improved.

**Lessons:**

Even though AS has a male predominance, clinicians should be aware that AS and TS may co-exist and that the clinical features are atypical in TS patients with AS.

## 1 Introduction

Turner syndrome (TS) is one of the most common sex chromosome abnormalities, affecting 1 in 2500 live-born females.^[1]^ Although the classical TS karyotype is monosomy X (45, X), part absence of the X chromosome or mosaicism occurs in about half of cases.^[2]^ Clinical manifestations include congenital lymphedema, short stature, gonadal dysgenesis, and an increased risk of other diseases such as autoimmune diseases.^[3]^

Ankylosing spondylitis (AS) is an autoimmune disease characterized by inflammatory arthritis of the spine to cause chronic back pain. AS affects males about twice as often as females^[4]^ and can have extra-articular manifestations such as acute anterior uveitis.^[5]^ Imaging and human leukocyte antigen-B27 (HLA-B27) testing are important diagnostic techniques because AS is often associated with characteristic radiological appearances and HLA-B27 positivity.

Co-existence of TS and AS is relatively rare. Here we present 2 such cases and take the opportunity to review the literature on the co-existence of these 2 disorders. Clinicians need to be aware that TS and AS may co-exist and have a high index of suspicion for AS in young female TS patients so that appropriate management can be started.

## 2 Case presentation

### 2.1 Case 1

A 13-year-old female patient was admitted to hospital with intermittent, bilateral hip joint pain. She had a 3-year history of pain on both sides of the hip joint that limited walking but that was partially relieved by exercise. There was no discomfort of the lower back or buttocks, and there were no symptoms of peripheral arthritis, enthesitis, or dactylitis.

**Figure 1:**
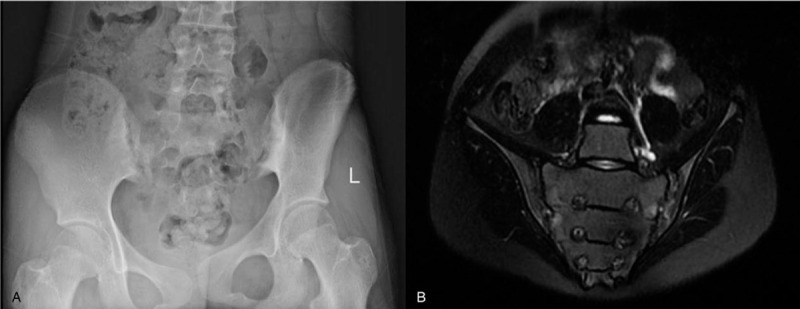
Case 1. (A) x-Ray showing a blurred surface of the sacroiliac joints bilaterally, with elevated density on the iliac side. (B) Magnetic resonance imaging showing coarse sacroiliac joint surfaces with disseminated patches of long T1 signals. The changes are consistent with ankylosing spondylitis.

Laboratory tests revealed an elevated erythrocyte sedimentation rate (ESR) and high-sensitivity C-reactive protein (hsCRP), and HLA-B27 was positive. Plain x-rays showed blurring of the articular surfaces of the sacroiliac joint, widening of the articular space, and swelling of the surrounding soft tissue, indicating grade 3 sacroiliitis (Fig. 1A). She had been diagnosed with juvenile AS at 10 years of age and was treated with sulfasalazine 0.5 g t.i.d. and diclofenac 25 mg t.i.d. for 1 year, which improved symptoms. However, these medications were stopped due to an elevated alanine aminotransferase. The hip pain and difficulty walking reappeared when she was 13.

She was diagnosed with TS at 9 years of age when she was seen in hospital for her nanism. Her karyotype was 45, X^[6]^/46, XX^[7]^. She was treated with growth hormone 3 U/day, which was suspended during exacerbations of AS. She had no manifestation of TS except for nanism, and she reached menarche at the age of 12.

Schober test was normal (7 cm), and Patrick sign was negative for both hips. There was, however, restricted adduction, abduction, and flexion of the hip joints bilaterally. There was also tenderness in the inguinal region.

Laboratory studies at this time revealed an ESR of 60 mm/h, hsCRP of 6 mg/L, and HLA-B27 positivity. Antinuclear antibodies (ANA), anti-double-stranded deoxyribonucleic acid (dsDNA) antibodies, and rheumatoid factor (RF) were negative. Magnetic resonance imaging of the sacroiliac joint showed typical changes of AS, which appeared to have progressed from the image taken 1 year previously (Fig. 1B).

She was diagnosed with a relapse of AS. Infection was excluded before starting immunosuppressive therapy. A purified protein derivative test for tuberculosis was, however, positive, and patchy linear opacities were visible in pulmonary computed tomography images. She was treated with tocilizumab 320 mg (8 mg/kg) once a month for 8 months and isoniazid and rifampicin for 3 months. At follow-up after 8 months, all symptoms had resolved and the ESR had dropped to 16 mm/h.

### 2.2 Case 2

**Figure 2:**
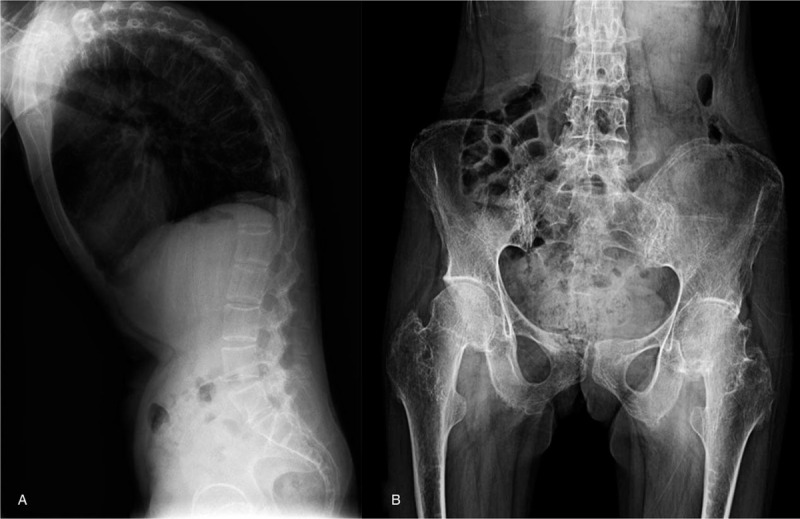
Case 2. (A) x-Ray showing thoracic kyphosis, squaring of the vertebral bodies, sclerosis of some vertebral body edges, blurred facet joints with partly disappearing joint spaces, and calcification of the anterior longitudinal ligament. (B) x-Ray showing blurred surfaces of the sacroiliac joints bilaterally.

A 27-year-old female patient attended hospital after developing thoracic kyphosis on the background of AS diagnosed at age 13. She had been treated with diclofenac, which was withdrawn after symptom relief. She had developed progressive thoracic kyphosis 2 years previously (Fig. 2A), but she experienced no lower back pain.

She had been diagnosed with TS at the age of 13 after being investigated for her short stature. Her karyotype was 45, X.

On admission, the patient showed gait characteristics of claudication. She was 124-cm tall (2 standard deviations below age- and sex-matched standards). She had a short neck without prominent webbing, cubitus valgus, a barrel chest, and no breast or nipple development. Patrick sign was positive on both sides. Internal and external rotation of the hip joints and knee flexion were limited. Her joints were not swollen. An x-ray showed blurred surfaces of the sacroiliac joints bilaterally (Fig. 2B).

Laboratory studies revealed an ESR of 68 mm/h and an hsCRP of 15.82 mg/L. HLA-B27 was positive and ANA, anti-dsDNA antibodies, anti-extractable nuclear antigen antibodies, and RF were negative. She underwent pedicle subtraction osteotomy of L1 via a posterior spinal approach, internal fixation of T10-L4, and bone graft fusion of the facet joint and vertebral plate of T10-L4. The operation was successful, and the patient was discharged 2 weeks after the operation. The patient recovered well during the 10-month follow-up.

## 3 Discussion

Peking Union Medical College Hospital treated 77 patients with TS between 1984 and 2018, two of whom also had AS. Although perhaps a coincidence, these 2 diseases may be associated.

The characteristics of these two patients and five other cases documented in the literature are listed in Table 1.^[6,8–11]^ The majority of reported patients had the monosomy X karyotype. All patients were positive for HLA-B27. Low back pain, a typical feature of AS,^[7]^ was only present in 3 of 7 patients. Osteoporosis was also present in 4 of 7 cases, with both diseases known to be associated with osteoporosis.^[12–15]^ Generally, TS patients with AS seem to present with relatively atypical features, and evaluation of bone mineral density should be carried out as part of the management given the association with osteoporosis. Moreover, AS developed at an early age in our patients, indicating the possibility of early onset in this context.

**Table 1:**
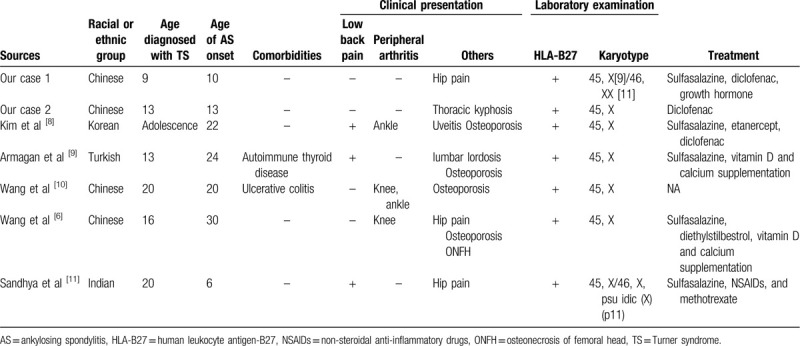
Characteristics of published cases of TS patients with AS.

It is widely accepted that TS is associated with increased risk of many autoimmune diseases including Hashimoto's thyroiditis, celiac disease, and inflammatory bowel disease. In a cohort study of 2459 TS patients in the UK, 4 TS patients had AS, with a rate ratio of 5.6 (95% confidence interval 1.5–14.4).^[16]^ Another Danish TS cohort divided autoimmune diseases according to their susceptibility by sex,^[17]^ and concluded that TS patients are at higher risk of autoimmune diseases, especially those with a male predominance. Unfortunately, these epidemiological studies reported few clinical details, precluding further interpretation of the TS-AS association. Attempts have been made to explore the mechanistic basis of autoimmune disease susceptibility in TS patients, with 1 study suggesting that susceptibility is related to haploinsufficiency of some X-linked genes.^[18]^ However, further work is required to establish the cause of this association.

Over half of TS patients have a 45, X mosaic karyotype.^[19,20]^ Mosaic patients usually do not have the typical manifestations seen with patients with monosomy X, so are at a higher risk of misdiagnosis.^[21]^ Nevertheless, early diagnosis and treatment are important for the prognosis of TS patients.^[22]^ For example, patients can reach normal heights if given growth hormone from 2 to 5 years of age. We believe that in female AS patients, TS—especially with mosaicism—may remain undiagnosed. However, these patients must be identified because TS patients are at higher risk of many clinical problems such as cardiovascular disease and metabolic disorders,^[23,24]^ which require follow-up and management. Moreover, some TS patients have elevated liver enzymes^[25]^ (as in case 1), and hepatotoxic medications should be avoided, not least in the management of AS in TS patients.

## Acknowledgments

The authors thank Dr. Hongbo Yang in the Department of Endocrinology and Dr. Min Shen in the Department of Rheumatology, Peking Union Medical College Hospital, for their assistance in the diagnosis and treatment of our patients.

## Author contributions

**Data curation:** Fang-Fei Chen, Xue-Han Zhang

**Funding acquisition:** Yang Jiao

**Writing – original draft:** Fang-Fei Chen

**Writing – review & editing:** Xue-Han Zhang, Yang Jiao
